# Spatial patterning and floral synchrony among trillium populations with contrasting histories of herbivory

**DOI:** 10.7717/peerj.782

**Published:** 2015-02-19

**Authors:** Christopher R. Webster, Michael A. Jenkins, Aaron J. Poznanovic

**Affiliations:** 1 School of Forest Resources and Environmental Science, Michigan Technological University, Houghton, MI, USA; 2 Department of Forestry and Natural Resources, Purdue University, West Lafayette, IN, USA; 3 Department of Forest Resources, University of Minnesota, St. Paul, MN, USA

**Keywords:** Herbivory, White-tailed deer, Trillium, Great Smoky Mountains National Park, Spatial pattern, Long-term monitoring

## Abstract

We investigated the spatial patterning and floral synchrony within and among populations of a non-clonal, forest understory herb, *Trillium catesbaei*. Two populations of *T. catesbaei* within Great Smoky Mountains National Park were monitored for five years: Cades Cove (high deer abundance) and Whiteoak Sink (low deer abundance). All individuals within each population were mapped during year one and five. Only flowering and single-leaf juveniles were mapped during intervening years. Greater distances between flowering plants (plants currently in flower) and substantially lower population densities and smaller patch sizes were observed at Cades Cove versus Whiteoak Sink. However, with the exception of flowering plants, contrasting histories of herbivory did not appear to fundamentally alter the spatial patterning of the *T. catesbaei* population at Cades Cove, an area with a long and well-documented history of deer overabundance. Regardless of browse history, non-flowering life stages were significantly clustered at all spatial scales examined. Flowering plants were clustered in all years at Whiteoak Sink, but more often randomly distributed at Cades Cove, possibly as a result of their lower abundance. Between years, however, there was a positive spatial association between the locations of flowering plants at both sites. Flowering rate was synchronous between sites, but lagged a year behind favorable spring growing conditions, which likely allowed plants to allocate photosynthate from a favorable year towards flowering the subsequent year. Collectively, our results suggest that chronically high levels of herbivory may be associated with spatial patterning of flowering within populations of a non-clonal plant. They also highlight the persistence of underlying spatial patterns, as evidenced by high levels of spatial clustering among non-flowering individuals, and the pervasive, although muted in a population subjected to chronic herbivory, influence of precipitation and temperature on flowering in long-lived forest herbs.

## Introduction

Spatial relationships within ecological communities increasingly are a focus of theoretical modelling efforts and empirical studies (Bacaro & Ricotta, 2007; Cain & Damman, 1997; Perry et al., 2008; Reine, Chocarro & Fillat, 2006; Zhang et al., 2010). Within plant populations, the spatial arrangement of individuals influences numerous density- and distance-dependent processes including pollination (Ison et al., 2014; Knight, 2003a; Morgan, Wilson & Knight, 2005; Steven et al., 2003), seed dispersal (Helenurm & Barrett, 1987), seedling recruitment and survival (Feldman & Morris, 2011; Tomimatsu & Ohara, 2002), and interspecific competition (Gustafsson & Ehrlén, 2003). Disturbances, such as herbivory, that have the potential to alter both the abundance and the spatial patterning of individuals (e.g., plants in flower) within populations may have cascading effects on vital rates (e.g., reproduction and seed dispersal) and population persistence (Knight, 2004; Thomson et al., 1996). Therefore, spatial patterning, when considered in conjunction with life history characteristics, is a critical element of population biology with important implications for species conservation.

Herbivory by forest ungulates is a pervasive disturbance in temperate forest ecosystems (Côté et al., 2004; Melis et al., 2006; Nomiya et al., 2003; Nuttle, Ristau & Royo, 2014; Russell, Zippin & Fowler, 2001). At the community level, abundant populations of native and exotic ungulates have been associated with homogenization of forest understories (Rooney, 2009; Rooney et al., 2004; Royo & Carson, 2006), declines in rare plant species (Darl Fletcher et al., 2001; McGraw & Furedi, 2005; Mysterud & Østbye, 2004), regeneration failures (Heinen & Currey, 2000; Tremblay, Huot & Potvin, 2007), altered developmental trajectories (Horsley, Stout & DeCalesta, 2003), and the success of invasive plant species (Knight et al., 2009; Webster et al., 2008). However, herbivore densities similar to those under which contemporary plant communities developed may have positive effects on the composition and diversity of communities (Cook-Patton, LaForgia & Parker, 2014; Royo et al., 2010). For individual species, the direct effects of herbivory vary with palatability, growth form, and resource allocation patterns (Whigham & Chapa, 1999; Wiegmann & Waller, 2006).

In species that produce a single annual flush of leaves, such as some members of the lily and arum families, loss of photosynthetic ability resulting from consumption of leaves and stems has been associated with subsequent decreases in physical stature and flowering (Balgooyen & Waller, 1995; Bierzychudek, 1984; Lubbers & Lechowicz, 1989; Rooney, 1997; Ruhren & Handel, 2000). The best example of this phenomenon results from the preferential browsing of large flowering plants in the genus *Trillium* by white-tailed deer (*Odocoileus virginianus* Zimmermann) (Anderson, 1994; Augustine & Frelich, 1998; Knight, 2003a; Knight, 2003b; Knight, 2004; Rooney & Gross, 2003; Rooney & Waller, 2001). Resultant changes in plant stature and likelihood of flowering reduce fecundity by decreasing the number of flowering plants, the production of ovules by individual flowering plants (smaller plants produce fewer ovules), and the efficiency of pollinators (Griffin & Barrett, 2002; Irwin, 2000; Knight, 2003a; Knight, 2003b; Knight, 2004). The abundance and spatial arrangement of seeds in myrmecochorous herbs, such as *Trillium*, in turn, influences dispersal (Smith, Forman & Boyd, 1989) and resultant spatial patterns (Jenkins & Webster, 2009; Webster & Jenkins, 2008). Consequently, through their influence on vital rates and plant abundance, browsers may prompt changes in spatially dependent processes, further exasperating the negative effects of acute herbivory on population structure and persistence.

Another potential consequence of selective herbivory may be a reduction in the synchrony of mass flowering events within and among plant populations. In spite of a great number of studies examining vital rates and demography for members of the genus *Trillium*, floral synchrony within and between populations has not been explored in detail. Two theories have been put forward to explain quasi-cyclic and synchronous reproductive events within and among populations of perennial plants: resource matching and resource switching (Kelly & Sork, 2002). Under the resource matching hypothesis, inter-annual variability in flowering and seed production is determined by synchrony in important environmental variables, such as precipitation and temperature (Piovesan & Adams, 2005; Rees, Kelly & Bjørnstad, 2002). Resource switching holds that flowering and seed production are primarily carbon-limited as evidenced by a stronger negative autocorrelation with prior productivity than correlation with relevant environmental variables (Lyles et al., 2009). Switching may be triggered by weather events (Kelly & Sork, 2002). However, if flowering and seed production simply vary in response to environmental conditions without any evidence of resource switching (accumulation or diversion of resources), then variation in seed crops would be considered “putative masting” (Kelly, 1994). Kelly (1994) notes that while many examples of masting fall within this category, its evolutionary/adaptive significance is unclear. If masting is a result predominantly of resource switching, selective herbivory of flowering and large non-flowering plants should reduce the synchrony between populations with contrasting rates of herbivory by reducing resources available for reallocation. The size of the event would also likely be reduced as fewer plants would be of reproductive size. Whereas, if masting is due to resource matching, then regional climate patterns should promote some degree of synchrony regardless of browsing history, assuming reproductive aged plants are present (Jenkins, Webster & Rock, 2007). These effects, however, may be lagged in non-clonal stage structured populations since they require reallocation of carbohydrates accumulated in the plant’s perennial rhizome during the previous growing season (Lubbers & Lechowicz, 1989). In this latter case, herbivory would influence the size of the event rather than the synchrony of its occurrence.

We examined stage structure and spatial patterning in two *Trillium catesbaei* Elliott (Liliaceae) populations with contrasting histories of herbivory. We followed these populations through time (five years) to see if selective herbivory of plants in flower altered the spatial patterning of flowering within populations or the synchrony of flowering. Our inquiry was limited to two sites in an effort to gain spatial and temporal information at greater extents than typically employed for point pattern analyses of forest herbs. Specifically, we hypothesized that the population exposed to chronic herbivory would display greater interplant distances and more random distributions of mature life stages, including flowering individuals. We also hypothesize that differences in stage structure would increase through time in response to reduced reproductive output resulting from direct and indirect effects of chronic herbivory.

## Materials and Methods

### Study area and species

Cades Cove, located entirely within Great Smoky Mountains National Park (GSMNP), is a gently undulating ∼2700 ha valley surrounded by mountains. A mosaic of open fields and woodlots has been maintained in the Cove by the National Park Service (NPS) for cultural and historic interpretation (Jenkins, Webster & Rock, 2007). This portion of GSMNP has a well-documented history of deer overabundance (see Griggs et al., 2006; Jenkins, Webster & Rock, 2007 for a detailed chronology). Following a series of irruptive cycles, contemporary estimates suggest deer densities remain high but have stabilized at a level below their historic peak of 43 deer km^−2^ during the late 1970s (Kinningham, 1980; Wathen & New, 1989). A smaller valley, Whiteoak Sink, which shares similar geology, soils, overstory composition, and pre-park land use history, has been used as a reference area for Cades Cove in previous studies (Jenkins, Webster & Rock, 2007; Webster & Jenkins, 2014; Webster, Jenkins & Rock, 2005). However, unlike Cades Cove, farmsteads in Whiteoak Sink were allowed to succeed to forest after creation of GSMNP. Because of this difference in management history, substantially fewer deer have been observed at Whiteoak Sink compared to Cades Cove over the last four decades since the NPS began monitoring deer numbers (Webster, Jenkins & Rock, 2005).

Soils at both sites consist largely of Lonon silty clay loams with small intermixed outcrops of limestone and sandstone and small areas of loamy colluvium over limestone and shale residuum (Soil Survey Staff , 2014). Forest structure and composition were similar at both sites (Table 1). Common overstory species included *Acer rubrum, Carya* spp., *Liriodendron tulipifera*, *Quercus alba*, and *Quercus rubra*, with scattered *Pinus strobus* and *Tsuga canadensis*. Common understory tree species included *Cornus florida*, *Ilex opaca*, and *T. canadensis*. Herbaceous-layer species found at both sites included *Maianthemum racemosum, Polygonatum biflorum, Smilax glauca, Smilax rotundifolia*, and *Viola sororia*. For a detailed comparison of the herbaceous layers at each site see Webster, Jenkins & Rock (2005).

**Table 1:** Overstory attributes within *Trillium catesbaei* mapping plots at Cades Cove and Whiteoak Sink, Great Smoky Mountains National Park, TN.

	Cades Cove	Whiteoak Sink
Mean DBH (cm)	21.2	20.3
Max DBH (cm)	77.2	54.0
Basal area (m^2^ ha^−1^)	34.8	33.2
Live trees ha^−1^	562.5	737.5
Snags ha^−1^	37.5	37.5
Snag basal area (m^2^ ha^−1^)	3.3	0.8

*Trillium catesbaei* is a non-clonal, long-lived, perennial herb that flowers in the spring, but remains photosynthetically active throughout the summer (Case & Case, 1997; Jenkins, Webster & Rock, 2007; Webster & Jenkins, 2014). The species is native to the southern Appalachians and occurs in mesic to dry-mesic oak-hardwood forests (Case & Case, 1997). We selected this species because it is the only *Trillium* species that can still be found in appreciable numbers in Cades Cove (Webster, Jenkins & Rock, 2005). Historical records indicate that large populations of other *Trillium* species were present within forested areas of Cades Cove soon after the creation of GSMNP, but these populations have largely disappeared as a result of persistently high white-tailed deer numbers in this portion of GSMNP (Bratton, Mathews & White, 1980). Jenkins, Webster & Rock (2007) found that *T*. *catesbaei* populations in Cades Cover displayed truncated age structures and flowering by younger and smaller plants compared to populations found at Whiteoak Sink.

All necessary permits were obtained for the described field study. Specifically, a permit was issued by Great Smoky Mountains National Park (GSMNP), United States Department of Interior National Park Service, Tennessee, USA (GRSM-2013-sc1-0867).

### Field techniques

*Trillium catesbaei* is a common component of the spring flora of mesic to dry-mesic oak-hardwood forests classified by NatureServe as *Quercus alba*—(*Quercus rubra, Carya spp*.)—Forest (White et al., 2003). To identify potential sampling areas, we compiled a list of suitable forest stands at Cades Cove and Whiteoak Sink using a digital elevation model and vegetation map based on the NatureServe classification scheme for GSMNP (Madden et al., 2004). Each candidate stand was then visited to confirm the presence of *T. catesbaei* populations. One stand at each site was then selected at random from this refined list. A 20 × 40 m plot was located to best capture the distribution of the *T. catesbaei* population at each site. Plot corners were marked with rebar. Since both sites had the same aspect (280°), the long axis of each plot was established parallel to the slope on a bearing of 190°. We adjusted the timing of our sampling each year in an effort to capture the period of peak flowering. Each year sampling was confined to a single week for logistical reasons and to provide a paired snapshot of each site at the same phenological stage. This approach, however, precluded a robust assessment of the fate and actual reproductive output of individuals. At Cades Cove, we observed deer browsing within the plot shortly after we completed our sampling in 2013.

To facilitate mapping, each 20 × 40 m plot was divided into eight 10 × 10 m sampling stations. The spatial location of each individual *T. catesbaei* plant was mapped with a Haglof DME sonic range finder (Haglof Inc., Madison, MS, USA) and a tripod-mounted Suunto sighting compass (Suunto USA Inc., Carlsbad, CA, USA). The life stage (non-flowering single-leaf, non-flowering three-leaf, or flowering three-leaf) of each individual was recorded along with its location (distance and azimuth). We mapped all individuals in 2009 and 2013, but only non-flowering single-leaf and flowering plants during intervening years (2010, 2011, and 2012; Figs. S1–S3).

**Figure 1:**
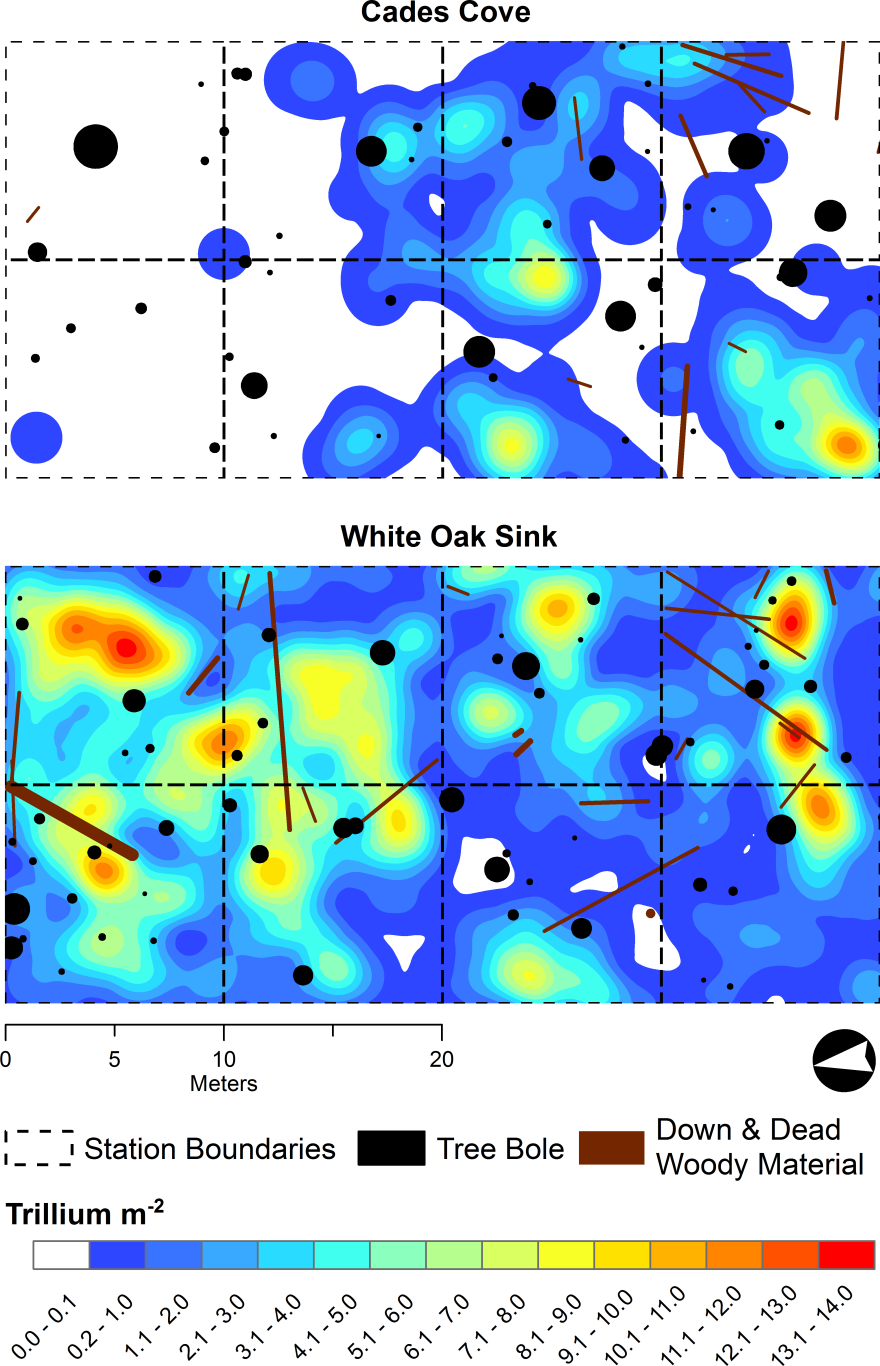
*Trillium catesbaei* populations and forest structure at start of study. Mapping plots at Cades Cove (historically high deer impact) and Whiteoak Sink (historically low deer impact) with locations of trees (≥2 cm diameter at breast height, 1.37 m) and coarse woody debris (≥10 cm diameter) overlaid on the 2009 *Trillium catesbaei* all stage kernel density interpolation.

Within each 20 × 40 m plot, we also mapped the location of all live and dead trees ≥5 cm diameter at breast height (DBH, 1.37 m), and recoded their species and DBH. All segments of down deadwood (DDW) ≥10 cm in diameter were also mapped (Fig. 1).

### Data analysis

Spatial analysis required converting field measurements of *T*. *catesbaei* locations to point spatial data in a GIS using ArcGIS 10.1^®^ software (ESRI, 2013). Additionally, we converted plot boundaries, tree locations, tree bole diameters at breast height, and DDW to vector spatial data. All spatial data were registered to the NAD 1983 UTM Zone 17N projected coordinate system.

We fit a smoothed raster surface (ESRI, 2013) to *T*. *catesbaei* point data by calculating the magnitude per unit area using a quadratic kernel function, following Silverman (1986) (p. 76, eq. (4.5)). The smoothed raster surface helped establish a visual relationship between *T. catesbaei* density at White Oak Sink and Cades Cove over a five year time period. The search radius was set to 2 m using a circular neighborhood, which provided a useful visual relationship of *T. catesbaei* spatial distributions.

Univariate point pattern analysis was conducted to test complete spatial randomness (CSR) of the *T. catesbaei* distributions. We assessed *T. catesbaei* spatial patterning in a multi-tiered approach first by study area, then by life stage and finally by year. The graphical output were summarized by distance *r* (m) based on the type of distribution. Univariate point pattern analysis using Besag’s transformation of the Ripley’s *K* function was implemented in the statistical computing environment R using the Lest function (Baddeley & Turner, 2005). Besag’s *L*-function takes the following form: $\hat {L}(r)=\sqrt{(\hat {K}(r)/\pi )}$, where $\hat {K}(r)=\frac{a}{n(n-1)}\sum _{i}\sum _{j}I({d}_{i j}\leq r){e}_{i j}$. In these equations, *r* refers to the search radius, *n* is the sample size, *i* and *j* are ordered point pairs, $I\left({d}_{i j}\leq r\right)$ indicates that if the distance is less than or equal to *r* let *I* = 1, *d_ij_* refers to the distance between *i* and *j*, and *e* is the edge correction method (Baddeley & Turner, 2005; Besag, 1977; Ripley, 1977). We used Besag’s *L* function because it is a variance stabilized transformation of the Ripley’s *K* function. The maximum search distance *r* was set at 5 m, following Ripley’s recommendation of using one-quarter of the smallest side of the analysis area (Baddeley & Turner, 2005). Ripley’s isotropic edge correction was used to reduce bias which results from unobservable points outside of the analysis window (Baddeley & Turner, 2005; Ripley, 1988). We determined through multiple trials that 15 was the minimum viable sample size. In order to test for CSR, we plotted the *L* function for the *T. catesbaei* point patterns with 3,000 simulations of CSR for the *L* function using the envelope function in R (Baddeley & Turner, 2005). From a graphical perspective, *T. catesbaei* point patterns deviating from CSR appear above the simulation envelope if clustered or below the simulation envelope if regularly distributed.

We also tested the bivariate point pattern of *T. catesbaei* between years using a cross-type *L* function. The algorithm was coded in R using the *L* cross function (Baddeley & Turner, 2005). The bivariate *L* function followed the form: ${L}_{i j}(r)=\sqrt{({K}_{i j}(r)/\pi )}$, where point *i* is assumed to be independent of point *j*. Similar to the univariate point pattern analysis, we also used 3,000 simulation of CSR and plotted the bivariate *L* function to test for CSR. All of the other parameters are identical to the univariate point pattern analysis. The results were displayed graphically and later summarized in tabular format depicting (a) a positive relationship between *i* and *j*; (b) a negative relationship between *i* and *j*; or (c) independence.

Mean nearest neighbor distance was calculated using the nndist function in R (Baddeley & Turner, 2005). The mean nearest neighbor calculates the distance to the first nearest neighbor in a point pattern. First nearest neighbor distance data were summarized first by study area and then within each study area by year and life stage.

The number of flowering plants during each year of the study were compared graphically to a cumulative estimate of spring (March 1–May 31) Palmer Drought Severity Index (PDSI). PDSI values were acquired from the National Oceanic and Atmospheric Admiration National Climatic Data Center (http://www.ncdc.noaa.gov/temp-and-precip/drought/historical-palmers.php).

## Results

**Figure 2:**
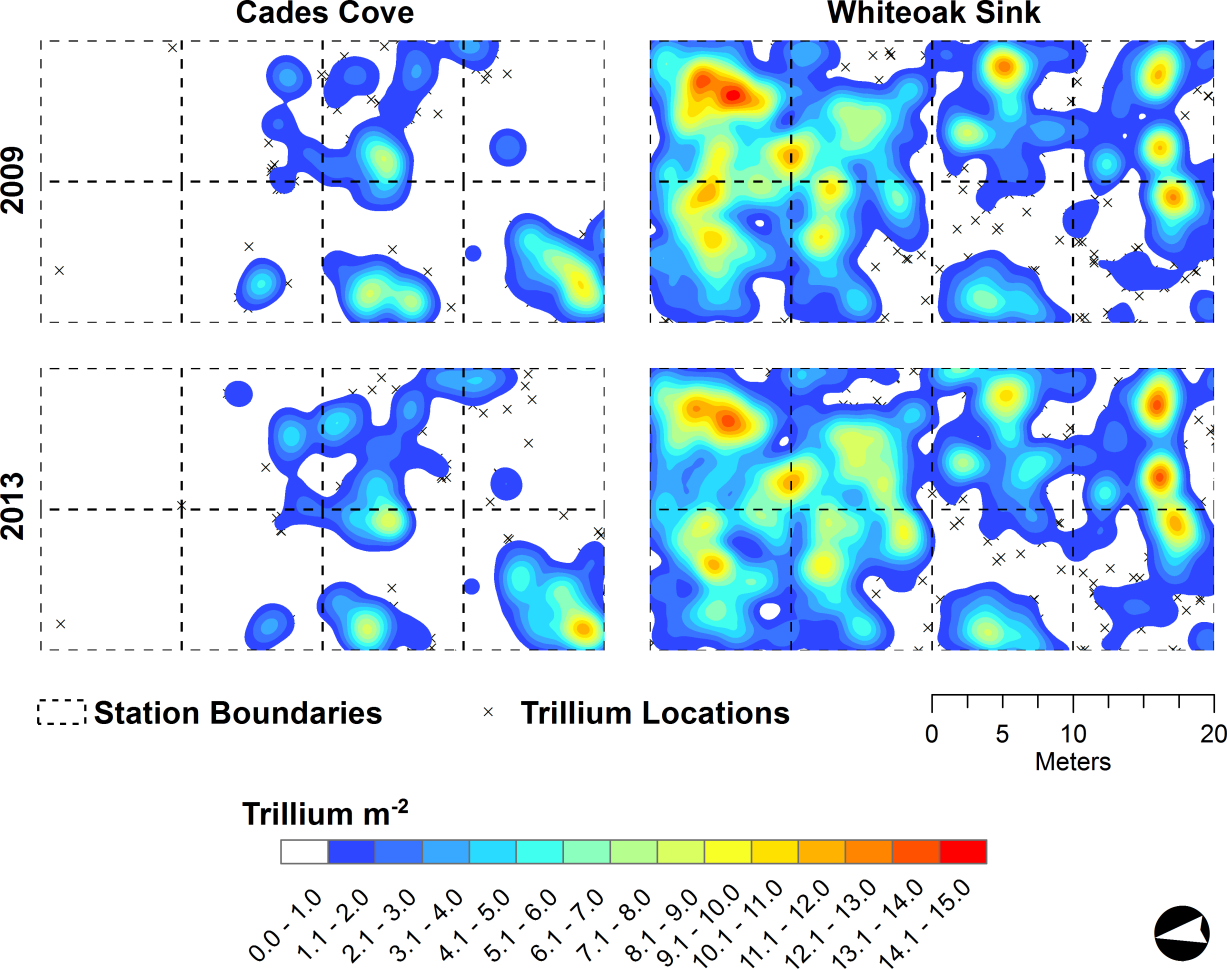
*Trillium catesbaei* all stage kernel density interpolations at the beginning (2009) and end of the study (2013) in mapping plots at Cades Cove (historically high deer impact) and Whiteoak Sink (historically low deer impact). Isolated T. catesbaei occurring at a density of less than one plant m^−2^ are denoted with an ‘x’.

Between 2009 and 2013 the number of individual *T. catesbaei* at both sites increased (Table 2). Patch coalescence and expansion was somewhat more pronounced at Whiteoak sink than Cades Cove, but in the aggregate (all stages combined) patch densities and boundaries at both sites were consistent through time (based on visual inspection of Fig. 2). Mean nearest neighbor distances for single-leaf and non-flowering three-leaf plants were similar between sites and years (Table 4). At both sites, the mean distance between flowering plants declined over time as the number of flowering individuals increased (Table 4). However, even at peak flowering in 2013, the mean distance between flowering plants at Cade Cove was an order of magnitude greater than that observed at Whiteoak Sink (1.64 ± 2.35 m versus 0.39 ± 0.34 m; Table 4).

**Table 2:** *Trillium catesbaei* demographics at Cades Cove and Whiteoak Sink, Great Smoky Mountains National Park, Tennessee.

	Number of plants	
Year/Life stage	Cades cove	Whiteoak sink
**2009**		
Single-leaf	147	384
Non-flowering three-leaf	469	2114
Flowering three-leaf	5	72
Total	621	2570
**2013**		
Single-leaf	107	402
Non-flowering three-leaf	554	1504
Flowering three-leaf	37	782
Total	698	2688
**Change between 2009 and 2013**		
Single-leaf	−40 (27.2%)	18 (4.7%)
Non-flowering three-leaf	85 (18.1%)	−610 (28.8%)
Flowering three-leaf	32 (640.0%)	710 (986.1%)
Total	77 (12.4%)	118 (4.6%)

**Figure 3:**
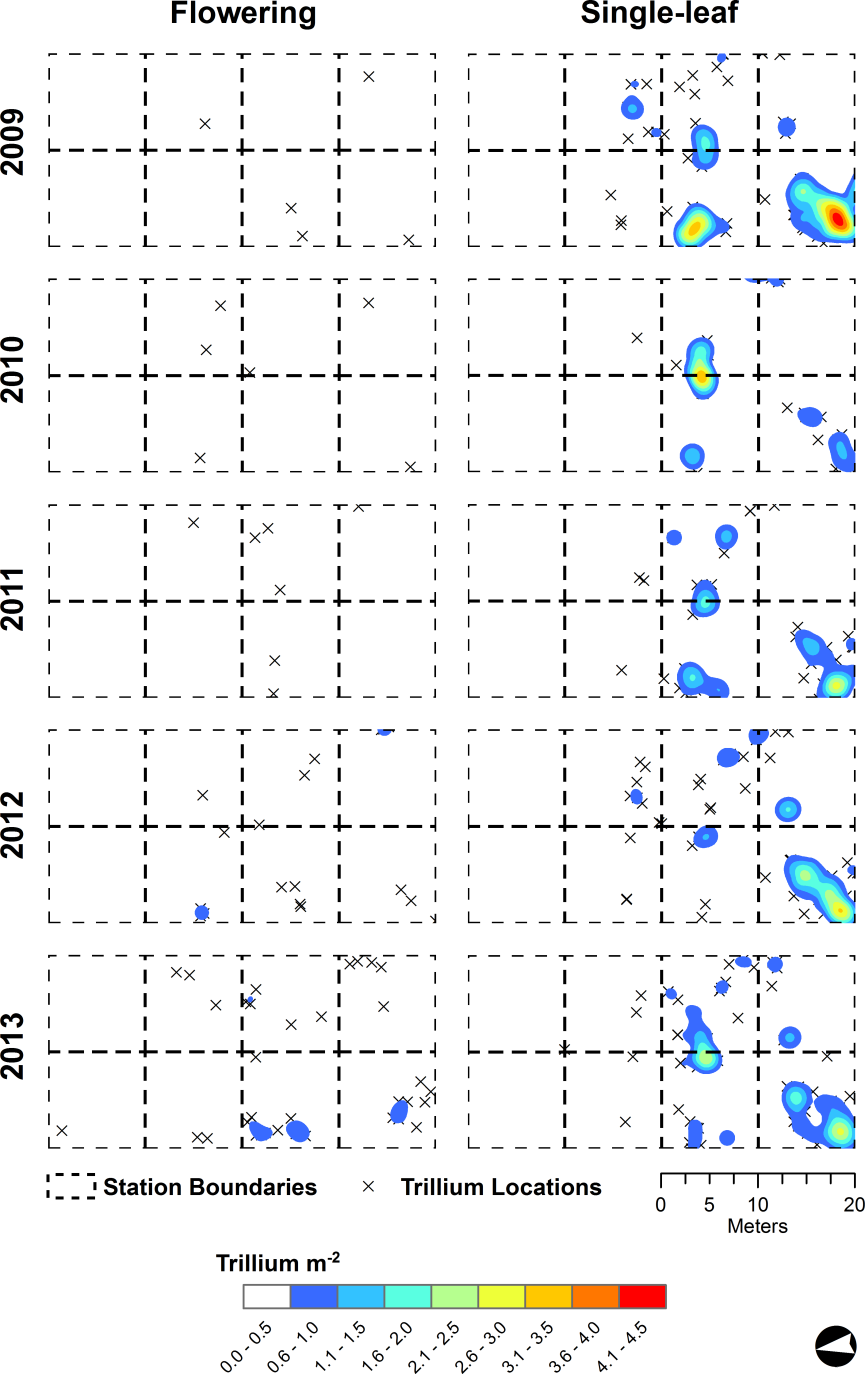
*Trillium catesbaei* kernel density interpolations at the Cades Cove (historically high deer impact) mapping plot for single-leaf and flowering plants. Isolated *T. catesbaei* occurring at a density of less than one plant m^−2^ are denoted with an ‘x.’

**Figure 4:**
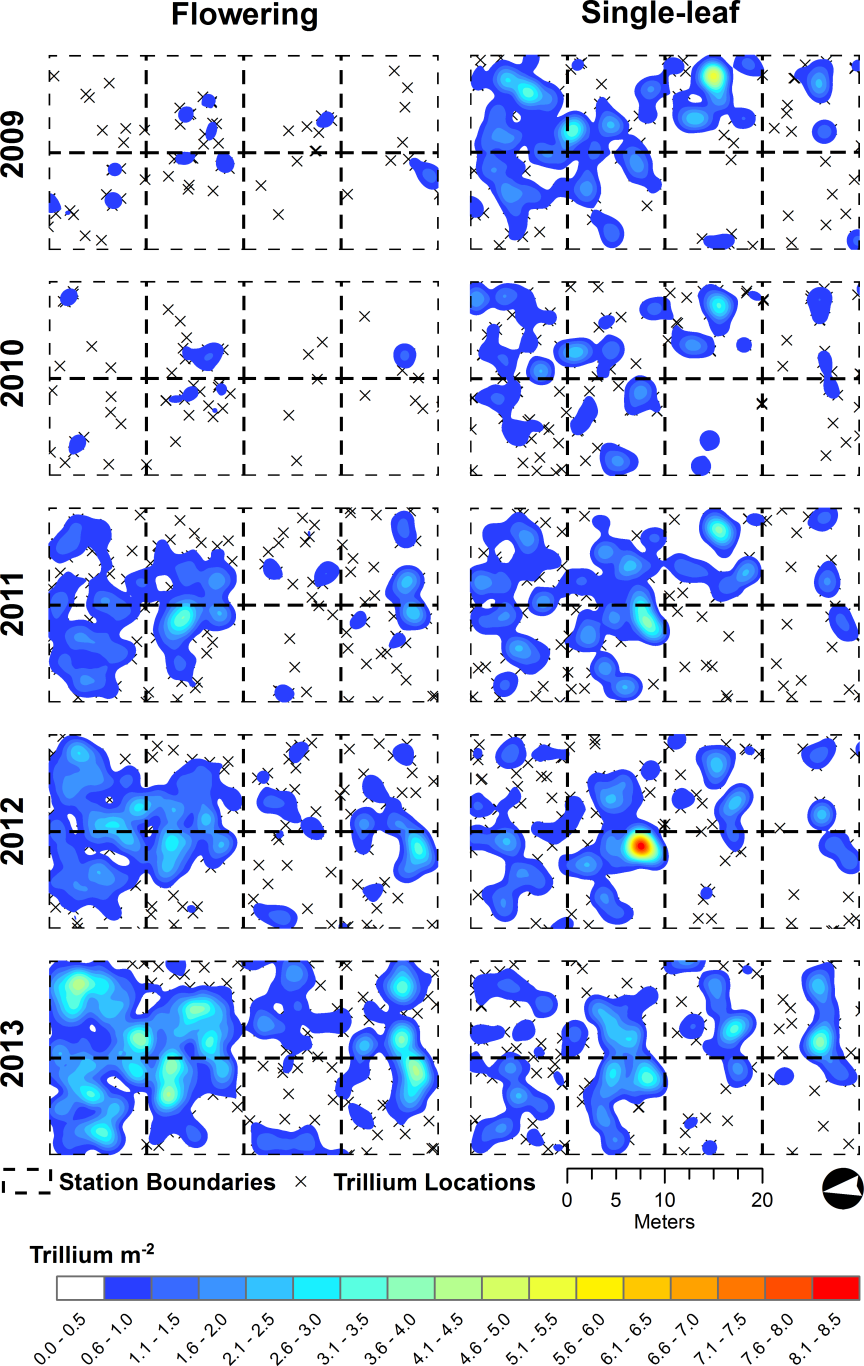
*Trillium catesbaei* kernel density interpolations at the Whiteoak Sink (historically low deer impact) mapping plot for single-leaf and flowering plants. Isolated *T. catesbaei* occurring at a density of less than one plant m^−2^ are denoted with an ‘x.’

All *T. catesbaei* life stages at Whiteoak Sink exhibited significant spatial aggregation at each of the spatial scales examined through time (Table 3). The same pattern was observed for single-leaf and non-flowering three-leaf plants at Cades Cove. Flowering plants, however, were too few in number at Cades Cove during the first three years of the study to evaluate spatial pattern. In 2012, as the number of flowering plants at Cades Cove more than doubled, the patterning of flowering plants was approximately random (Table 3). As the number of flowering plants again more than doubled in 2013, significant clustering was observed at all but the finest spatial scale investigated at Cades Cove (Table 3 and Fig. 3). Flowering plants at Whiteoak Sink were significantly clustered at each spatial scale through time irrespective of a nearly 10-fold increase in the number of flowering plants over the course of the study (Tables 2, 3 and Fig. 4).

**Table 3:** Univariate *L*(*r*) neighborhood analysis of spatial patterning of various *Trillium catesbaei* life history stages at Cades Cove and Whiteoak Sink.

	Cades Cove						Whiteoak Sink					
		Distance r (m)					Distance *r* (m)				
Year	*n*	1	2	3	4	5	*n*	1	2	3	4	5
**Single-leaf**												
2013	107	c	c	c	c	c	402	c	c	c	c	c
2012	96	c	c	c	c	c	367	c	c	c	c	c
2011	77	c	c	c	c	c	376	c	c	c	c	c
2010	55	c	c	c	c	c	247	c	c	c	c	c
2009	147	c	c	c	c	c	384	c	c	c	c	c
**Non-flowering three-leaf**												
2013	554	c	c	c	c	c	1504	c	c	c	c	c
2012	–	NA					–	NA				
2011	–	NA					–	NA				
2010	–	NA					–	NA				
2009	469	c	c	c	c	c	2114	c	c	c	c	c
**Flowering three-leaf**												
2013	37	x	c	c	c	c	782	c	c	c	c	c
2012	16	x	x	x	x	x	465	c	c	c	c	c
2011	7	NA					341	c	c	c	c	c
2010	6	NA					63	c	c	c	c	c
2009	5	NA					72	c	c	c	c	c

**Notes.**

Notes c significant (*α* = 0.05) spatial clustering u regular distribution x pattern did not differ significantly from random NA sample size insufficient

**Table 4:** Neighborhood attributes of various *Trillium catesbaei* life history stages at Cades Cove and Whiteoak Sink.

Cades Cove					Whiteoak Sink				
		Nearest neighbor distance				Nearest neighbor distance		
Year	*n*	Max. (m)	Mean (m)	Std. (m)	Year	*n*	Max. (m)	Mean (m)	Std. (m)
**Single-leaf**									
2013	107	7.10	0.64	1.10	2013	402	4.18	0.43	0.52
2012	96	3.36	0.55	0.69	2012	367	4.04	0.42	0.51
2011	77	4.45	0.50	0.67	2011	376	4.20	0.48	0.58
2010	55	4.91	0.46	0.79	2010	247	4.22	0.53	0.67
2009	147	5.80	0.45	0.72	2009	384	2.79	0.42	0.48
**Non-flowering three-leaf**									
2013	554	1.91	0.23	0.25	2013	1504	2.73	0.24	0.25
2012	NA				2012	NA			
2011	NA				2011	NA			
2010	NA				2010	NA			
2009	469	2.82	0.30	0.71	2009	2114	1.69	0.19	0.20
**Flowering three-leaf**									
2013	37	14.0	1.6	2.3	2013	782	2.26	0.39	0.34
2012	16	7.5	2.2	2.0	2012	465	4.47	0.49	0.47
2011	7	9.6	4.6	2.9	2011	341	3.65	0.61	0.55
2010	6	17.5	9.4	5.5	2010	63	5.08	1.40	1.28
2009	5	15.8	9.1	5.7	2009	72	5.49	1.09	1.04

Bivariate spatial analyses indicated that the locations of flowering plants during any given year were significantly associated with the location of flowering plants during the preceding year (Table 5). This relationship was consistent between years at Whiteoak Sink. However, at Cades Cove, sufficient densities for analysis were only available in 2012–2013. The locations of flowering plants in Cades Cove in 2013 were significantly associated with flowering locations in 2012.

**Table 5:** Bivariate *L*(*r*) neighborhood analysis of flowering *Trillium catesbaei* locations between years. A ‘+’ indicates a significant (*α* = 0.05) positive spatial association between individuals between sample years; NA indicates sample size was insufficient to analyze point patterns.

		Distance *r* (m)				
Years	*n*	1	2	3	4	5
**Cades cove**						
2013, 2012	37, 16	+	+	+	+	+
2012, 2011	16, 7	NA				
2011, 2010	7, 6	NA				
2010, 2009	6, 5	NA				
**Whiteoak Sink**						
2013, 2012	782, 465	+	+	+	+	+
2012, 2011	465, 341	+	+	+	+	+
2011, 2010	341, 63	+	+	+	+	+
2010, 2009	63, 72	+	+	+	+	+

**Figure 5:**
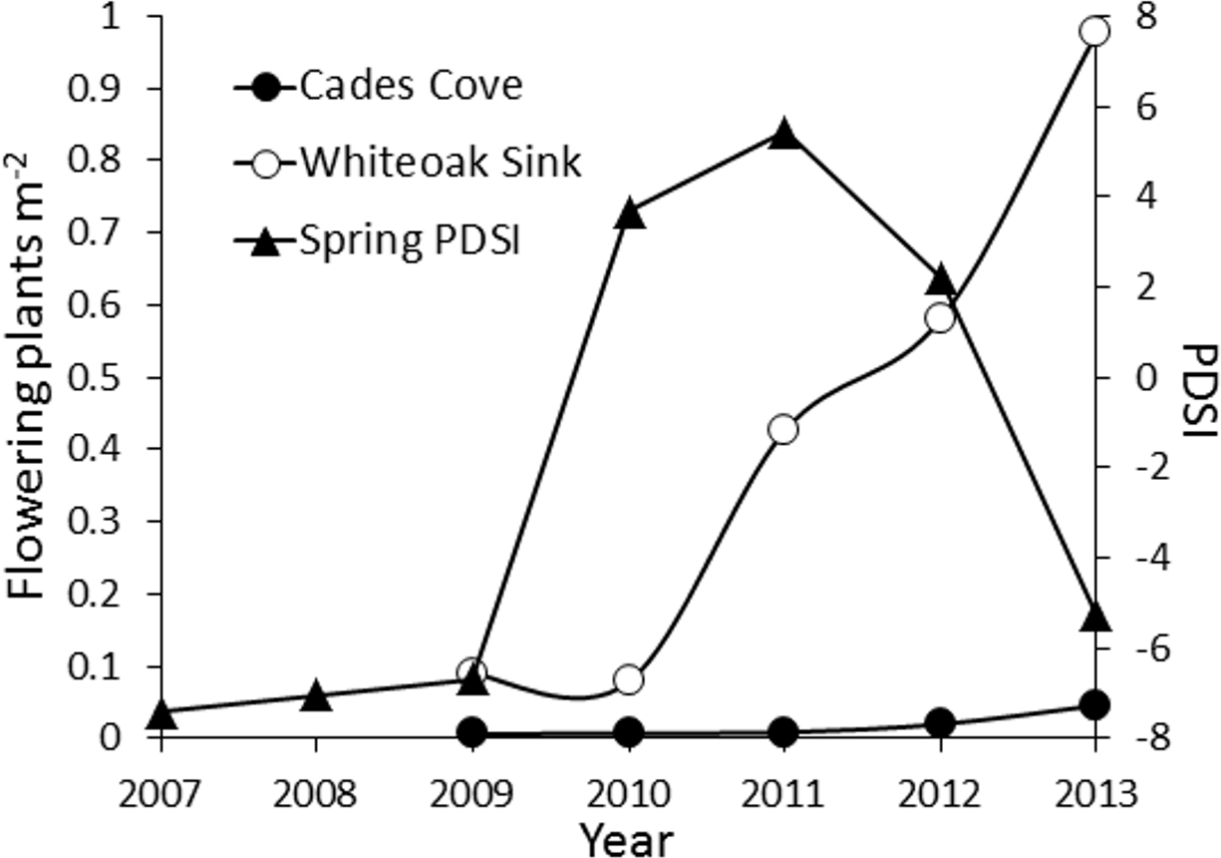
Flowering is higher in years following favorable spring climate conditions. Relationship between the number of flowering plants in the *Trillium catesbaei* populations at Cased Cove and Whiteoak Sink and spring (March 1–May 31) Palmer Drought Severity Index (PDSI) values over the course of the study. The 2013 PDSI value is from March 1 to the date of sampling (April 20, 2013).

Changes in the number of flowering plants at both sites over the course of our study appeared to be somewhat synchronous (Figs. 3 and 4). The density of flowering plants displayed evidence of a one-year lagged response to spring PDSI (Fig. 5). The first year of the study that did not experience a spring drought (negative PDSI) was 2009. The following spring (2010) Cades Cove and Whiteoak Sink experienced a 17 and 441% increase in flowering, respectively. Favorable spring moisture conditions during 2010 and 2011 were also followed by substantial increases in the number of flowering plants (Fig. 5). The greatest annual increase in number of flowering plants at Cades Cove was between 2012 and 2013 (131%), whereas the greatest increase at Whiteoak Sink was between 2010 and 2011 (441%).

## Discussion

It has long been recognized that high levels of white-tailed deer herbivory alter the abundance of life-stages within *Trillium* populations (e.g., Anderson, 1994; Knight, 2003b; Rooney & Gross, 2003) and threaten their long-term persistence (Jenkins, Webster & Rock, 2007; Knight, 2004). Our results shed additional light on the legacy of herbivory on the spatial patterning of life stages within populations of non-clonal herbs. They also highlight the persistence of underlying spatial patterns that commonly emerge as the result of dispersal limitation and resource heterogeneity (i.e., spatial clustering) and the pervasive influence of climate on flowering in long-lived forest herbs.

Visual examination of density maps for all stages combined suggests only minor patch recession, expansion, and coalescence through time, with few obvious differences associated with browse history. These maps, however, do suggest a good deal of internal dynamism associated with population increases at both sites. Cades Cove had a lower absolute density but higher percentage increase in population size compared to Whiteoak Sink. Changes within life stage appeared to be associated with transitions to more mature stages and the legacy of herbivory. Cades Cove experienced increases in non-flowering and flowering three-leaf plants, but a large (27%) reduction in the number of single-leaf plants in the population. Previous research at this site found that single-leaf plants were significantly older than those observed at Whiteoak Sink (Webster & Jenkins, 2014). In fact, many were old enough to have been reproductively mature (Jenkins, Webster & Rock, 2007; Webster & Jenkins, 2014). Under favorable environmental conditions, individuals residing in this class may have transitioned to non-flowering three-leaf and then flowering plants. Consequently, the changes in stage structure we observed at the Cades Cove mapping site suggest at least the possibility of a fledgling recovery of this *T. catesbaei* population; however, continued long-term monitoring will be necessary to establish this trend.

Significant spatial clustering across spatial scales was observed through time for single-leaf and non-flowering three-leaf *T. catesbaei* at both Cades Cove and Whiteoak Sink. In spite of substantial differences in density between Cades Cove and Whiteoak Sink (Table 2), mean nearest neighbor distances over the course of our study were surprisingly similar for both single-leaf (0.52 ± 0.07 versus 0.46 ± 0.05) and non-flowering plants (0.27 ± 0.03 versus 0.22 ± 0.03). Spatial clustering in non-clonal species generally arises in heterogeneous environments where suitable microsites or resources are limiting but competition between individuals is weak (Beatty, 1984; Maslov, 1989). In the genus *Trillium*, limited seed dispersal (Beattie & Culver, 1981; Handel, Fisch & Schatz, 1981) and dispersal interference (Kalisz et al., 1999; Ohara & Higashi, 1987; Yamagishi, Tomimatsu & Ohara, 2007) may also contribute to the high degree of spatial aggregation observed among members of this genus (Kawano, Ohara & Utech, 1992; Walker, Fore & Collins, 2009; Webster & Jenkins, 2008). Consequently, our results support that while herbivory may reduce the size and number of patches, underlying spatial patterns arising from other processes and patterns may persist even in communities otherwise degraded by herbivory.

In contrast to non-flowering plants, significant differences in the spatial arrangement of flowering plants were observed between Cades Cove and Whiteoak Sink. Consistent with our hypothesis, flowering plants at Cades Cove were an order of magnitude further away from their nearest neighbors than flowering plants at Whiteoak Sink. Additionally, during 2012 and 2013 (the only years with a sufficient sample size for analysis of flowering plant point patterns at Cades Cove) flowering plants were randomly distributed at a radius ≤ 1 m. In 2012, flowering plants were randomly distributed across all spatial scales. Flowering plants at Whiteoak Sink, on the other hand, were significantly clustered across all years and spatial scales. Random spatial patterns are typically associated within homogenous environments or spatially random chance events that limit establishment or result in mortality (Maslov, 1989; Miller, Mladenoff & Clayton, 2002). Additionally, low numbers of flowering plants may reduce the accuracy of pattern estimation resulting in random distributions in some cases (Ward & Ferrandino, 1999; Wolf, 2005). Small sample sizes tend to increase the size of the simulation envelope (i.e., confidence interval) making it difficult to distinguish patterns from random spatial distributions. Consequently, these data may be interpreted as evidence that herbivory is a spatially random process or simply reduces density to a point where patterns become indistinguishable from random.

Greater distances between flowering individuals may influence pollinator behavior and seed dispersal. Research by Knight (2003b), Knight (2004) with *Trillium grandiflorum* found that pollen limitation was common in browsed populations where densities of flowering plants were low. Similarly, Tomimatsu & Ohara (2002) observed limited seed production in fragmented *Trillium camschatcense* populations with <50 flowering plants. At low floral densities pollinators may switch to other species and deliver less conspecific pollen (Knight, 2004). Also, pollinator activity in early spring when *Trillium* species are in flower is unreliable and flowers often only receive a single pollinator visit (Griffin & Barrett, 2002). In addition, at low floral densities, increasing the number of neighboring flowering plants may enhance pollen delivery; but at high floral densities, neighboring plants became competitors for the limited number of visiting pollinators (Steven et al., 2003). Dispersal rates in ant-dispersed genera, such as *Trillium*, are similarly influenced by seed abundance (Smith, Forman & Boyd, 1989). Consequently, it seems likely that through its influence on the abundance and patterning of flowering plants, deer herbivory may both directly (selective consumption of flowering plants) and indirectly (greater likelihood of pollen and dispersal limitation) alter reproductive performance of individual plants and populations.

We observed evidence of floral synchrony among *T. catesbaei* populations at both Cades Cove and Whiteoak Sink. The number of flowering plants in both populations increased steadily from 2010 to 2013, and visually appeared to lag a year behind favorable spring growing conditions (Fig. 5). Large flowering events in some species, such as *Goodyera pubescens*, follow years with warm dry springs (Reddoch & Reddoch, 2007). A partial resurvey during 2014 confirmed that flowering declined in response to the dry spring of 2013 as would be expected from the trend in Fig. 5. Also, the location of flowering plants within populations was autocorrelated with the location of flowering plants the previous year. These findings provide tentative support for resource matching as a driver of floral synchrony among these populations. Herbivory reduced the size of the event at Cades Cove relative to Whiteoak Sink, but did not reduce its synchrony. Flowering in many *Trillium* species is size dependent with larger flowering and non-flowering plants more likely to flower in subsequent years (Anderson, 1994; Knight, 2003a; Lubbers & Lechowicz, 1989; Rooney & Waller, 2001). However, previous research at our study sites suggests that plants may flower when younger and smaller at Cades Cove than Whiteoak Sink (Jenkins, Webster & Rock, 2007). This suggests that “mature” plants allocate resources to subsequent flowering during years of favorable environmental conditions and may remain in the flowering stage as long as conditions remain suitable for allocating current year photosynthate to subsequent flowering. Prolonged periods of synchronous flowering are consistent with the observation that animal-pollinated species dispersed by mutualistic frugivores should be less variable in their reproductive effort (Herrera et al., 1998). Masting may also facilitate dispersal by satiating seed predators (Vander Wall, 2002); however, in the case of *T. catesbaei*, while mass seeding may satiate some seed predators, it is unlikely that mass flowering satiates foraging deer, especially if episodes are multi-year (Kelly & Sork, 2002). The recent increase in population size at Cades Cove, however, suggests that during these comparably large floral displays, at least a few plants are escaping herbivory long enough to produce seed in a population that has otherwise experienced little successful reproduction in decades (Jenkins, Webster & Rock, 2007). Nevertheless, it remains possible that the mass flowering event we observed was simply putative masting in response to favorable environmental conditions. The level of resource switching associated with these events clearly warrants further investigation.

While the inferences that can be drawn from two sites are somewhat limited, this study provides a useful data point for broader inquiries into the spatial and temporal dynamics of forest herbs. Furthermore, the level of temporal detail regarding the spatial distributions of various life stages, particularly flowering plants, provides a baseline as these forest understories respond to exogenous disturbances and environmental change.

## Supplemental Information

10.7717/peerj.782/supp-1 Figure S1 Spatial locations of all life stages of *Trillium catesbaei* at the beginning (2009) and end of the study (2013)

10.7717/peerj.782/supp-2 Figure S2 Spatial locations of *Trillium catesbaei* within the Cades Cove (historically high deer impact) mapping plot for single-leaf and flowering plants

10.7717/peerj.782/supp-3 Figure S3 Spatial locations of *Trillium catesbaei* within the Whiteoak Sink (historically low deer impact) mapping plot for single-leaf and flowering plants

## Additional Information and Declarations

### Field Study Permissions

The following information was supplied relating to field study approvals (i.e., approving body and any reference numbers): Great Smoky Mountains National Park. Permit #: GRSM-2013-sc1-0867
