# Supplementary figures and images for: Spatial patterning and floral synchrony among trillium populations with contrasting histories of herbivory
Source: PeerJ. 2015 Feb 19;3:e782. doi: 10.7717/peerj.782 (PMC4338797; doi:10.7717/peerj.782)

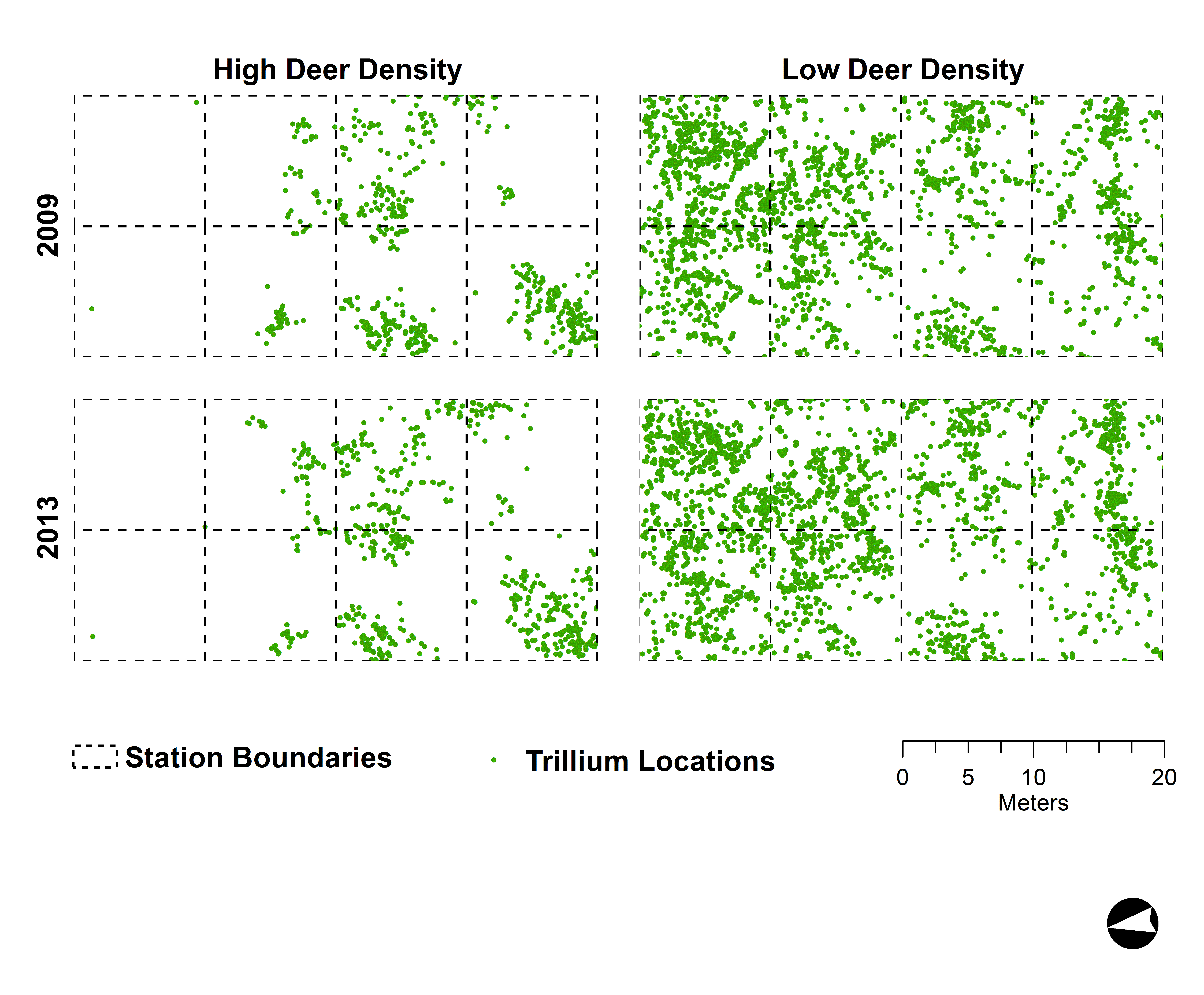

Supplement: Figure S1 [file peerj-03-782-s001.png]

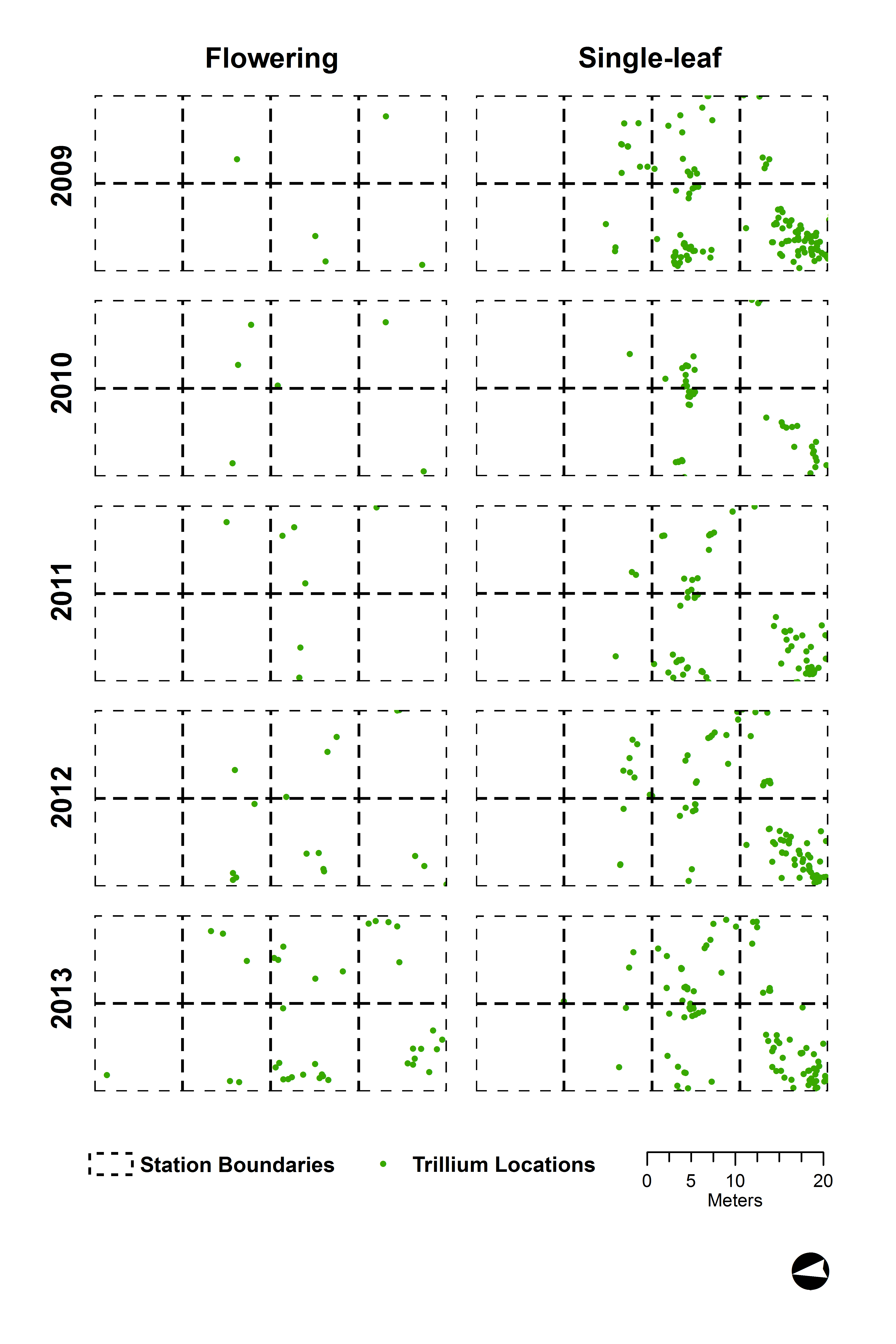

Supplement: Figure S2 [file peerj-03-782-s002.png]

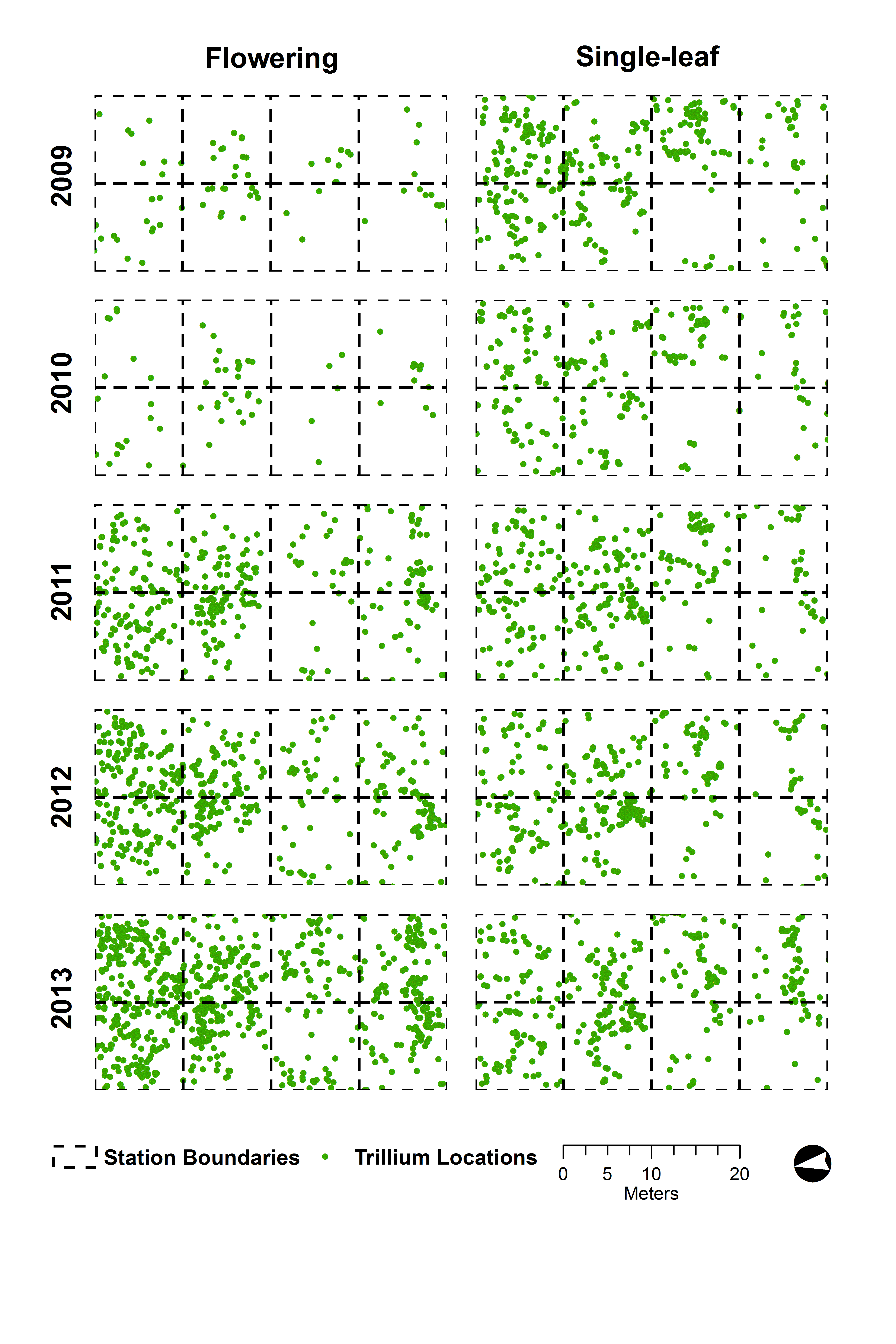

Supplement: Figure S3 [file peerj-03-782-s003.png]
